# Identification of the novel deletion-type *PML-RARA* mutation associated with the retinoic acid resistance in acute promyelocytic leukemia

**DOI:** 10.1371/journal.pone.0204850

**Published:** 2018-10-05

**Authors:** Hikaru Hattori, Yuichi Ishikawa, Naomi Kawashima, Akimi Akashi, Yohei Yamaguchi, Yasuhiko Harada, Daiki Hirano, Yoshiya Adachi, Kotaro Miyao, Yoko Ushijima, Seitaro Terakura, Tetsuya Nishida, Tadashi Matsushita, Hitoshi Kiyoi

**Affiliations:** 1 Department of Hematology and Oncology, Nagoya University Graduate School of Medicine, Nagoya, Japan; 2 Department of Medical Technique, Nagoya University Hospital, Nagoya, Japan; 3 Department of Transfusion Medicine, Nagoya University Hospital, Nagoya, Japan; European Institute of Oncology, ITALY

## Abstract

All-*trans* retinoic acid (ATRA) and arsenic trioxide (ATO) are essential for acute promyelocytic leukemia (APL) treatment. It has been reported that mutations in *PML-RARA* confer resistance to ATRA and ATO, and are associated with poor prognosis. Although most *PML-RARA* mutations were point mutations, we identified a novel seven amino acid deletion mutation (p.K227_T233del) in the *RARA* region of *PML-RARA* in a refractory APL patient. Here, we analyzed the evolution of the mutated clone and demonstrated the resistance of the mutated clone to retinoic acid (RA). Mutation analysis of *PML-RARA* was performed using samples from a chemotherapy- and ATRA-resistant APL patient, and the frequencies of mutated *PML-RARA* transcript were analyzed by targeted deep sequencing. To clarify the biological significance of the identified *PML-RARA* mutations, we analyzed the ATRA-induced differentiation and PML nuclear body formation in mutant *PML-RARA*-transduced HL-60 cells. At molecular relapse, the p.K227_T233del deletion and the p.R217S point-mutation in the RARA region of *PML-RARA* were identified, and their frequencies increased after re-induction therapy with another type of retinoiec acid (RA), tamibarotene. In deletion *PML-RARA*-transduced cells, the CD11b expression levels and NBT reducing ability were significantly decreased compared with control cells and the formation of PML nuclear bodies was rarely observed after RA treatment. These results indicate that this deletion mutation was closely associated with the disease progression during RA treatment.

## Introduction

Acute promyelocytic leukemia (APL) is characterized by the chromosomal translocation t(15;17)(q22;q21), which results in *PML-RARA* fusion transcript [1] [2]. APL with *PML-RARA* is categorized into the a favorable risk group of acute myeloid leukemia (AML) with a higher complete remission rate over 90% and an overall survival rate of over 80% at 5 years [3]. Combination therapy with anthracycline-based chemotherapy and all-*trans* retinoic acid (ATRA) has markedly improved the clinical outcome of APL patients compared with conventional chemotherapy alone [4–6]. Subsequently, the introduction of arsenic trioxide (ATO) has also further improved clinical APL therapy[7–9]. The efficacy and safety of front-line ATRA and ATO combination therapy omitting chemotherapeutic agents has been demonstrated in several clinical studies, and can be a standard front-line therapy in low- to intermediate-risk APL patients. Additionally, tamibarotene, a synthetic retinoic acid (RA), has also been developed to overcome ATRA resistance and exhibits clinical efficacy [10, 11]. Thus, RA and ATO are indispensable for the treatment of APL.

However, some patients still develop resistance to RA and/or ATO. It is known that mutations in *PML-RARA* confer resistance to RA and ATO, and are associated with poor prognosis in APL patients [12, 13]. Reportedly, mutations in *PML-RARA* were identified in about 30% and 60% of APL patients at the first and second relapse, respectively [14]. Although most acquired mutations associated with RA resistance were point mutations in *PML-RARA*, we identified a novel seven amino acid deletion mutation (p.K227_T233del) as well as a point mutation, p.R217S, in the *RARA* region of *PML-RARA* from an RA-resistant APL patient; however, the biological significance of these mutations was not analyzed. Here we report on the clinical and biological significance of the deletion mutation in the *RARA* region of *PML-RARA*. We demonstrated that the deletion mutation was critical for RA sensitivity, whereas p.R217S did not affect the sensitivity *in vitro*. Moreover, we found that RA-induced PML-nuclear body (NB) formation was affected in the APL cells harboring the deletion mutation. Our results indicate that this acquired deletion mutation in *PML-RARA* was a critical mechanism for the acquisition of resistance to RA therapy, and thus that it is necessary to evaluate the biological significance of such mutations to improve clinical strategies for relapse/refractory APL patients.

## Materials and methods

### Patient and samples

The patient complained of posterior cervical tumor and was diagnosed as myxofibrosarcoma (MFS) by biopsy at the age of 26. She then received a proton radiation therapy, and chemotherapy was administrated for total two years as follows: two cycles of a combination of ifosfamide (IFO) and doxorubicine (DXR), three cycles of a combination of ifosfamide, carboplatin (CBDCA) and etoposide (VP-16), and subsequently 13 cycles of a combination of carboplatin and etoposide. After three months of the last chemotherapy, when the patient had achieved complete remission (CR) of MFS, she complained of an atypical genital bleeding and revealed leukocytosis and thrombocytopenia. The diagnosis of APL was obtained from bone marrow examination based on the morphology and expression of the *PML-RARA* fusion transcript [15, 16]. After diagnosis, the patient immediately received ATRA, and combination chemotherapy with idarubicin (IDR) and cytarabine (AraC) was administered because her white blood cell count was over 10,000/μl at diagnosis, leading to the first CR of APL. Subsequently, consolidation therapy with ATO was initiated, but it was discontinued at day 15 due to whole body muscle pain. Consolidation therapy was switched to conventional chemotherapy with mitoxantrone (MIT) and cytarabine; however, *PML-RARA* transcript emerged in the hematological recovery phase at two consecutive points, and we diagnosed the patient with molecular relapse of APL. Re-induction therapy with the other RA, tamibarotene, was initiated, whereas the total amount of *PML-RARA* transcript increased, and hematological relapse was revealed after high-dose cytarabine (HiDAC) therapy subsequent to tamibarotene. The patient then underwent chemotherapy and allogeneic hematopoietic stem cell transplantation (allo-HSCT) after hematological relapse. Although the patient achieved molecular remission one month after the allo-HSCT, the *PML-RARA* transcript was increased four months later and exhibited further relapse after the allo-HSCT.

Bone marrow (BM) samples from the patient were subjected to Ficoll-Hypaque density gradient centrifugation. For the germ-line control, buccal swabs or PB mononuclear cells (MNCs) were collected during the disease-free period. We obtained written informed consent from the patient to use sequential samples for banking and molecular analysis, and approval of the study was obtained from the ethics committee of Nagoya University School of Medicine.

### Cell lines and cell culture

The human promyelocytic leukemia cell lines NB4 and HL-60 were maintained in RPMI1640 medium (Invitrogen, Carlsbad, CA) with 10% FCS.

### Reagents

ATRA, tamibarotene and ATO were purchased from Sigma-Aldrich (St. Louis, MO).

### Mutation analysis

Total RNA was extracted using the QIAamp RNA Blood Mini Kit (QIAGEN, Hilden, Germany) and reverse transcribed using the SuperScript II reverse transcriptase (Thermo Fisher Scientific, Waltham, MA) according to the manufacturer’s instructions. *PML-RARA* chimeric transcript were amplified from each cDNA by reverse transcriptase-mediated PCR and mutations in the ligand binding domain (LBD) were examined by the direct-sequencing method using the forward primer, 5'-ACCTCAGCTCTTGCATCACC-3', and reverse primer, 5'-TTGAGGAGGGTGATCTGGTC-3'. To confirm each mutation, we used cloning procedures. The PCR-amplified 637-base pair fragments of the *PML-RARA* gene products were separated on agarose gel and purified using the QIAquick Gel Extraction Kit (QIAGEN). Each purified fragment was cloned into the pGEM-T Easy vector (Promega), and then transfected into *Escherichia coli* strain DH5α. Recombinant colonies were randomly selected from PCR-amplified libraries, and plasmid DNA was prepared using the QIAprep Spin Miniprep Kit (QIAGEN) and sequenced. We also investigated the mutant allele burden of these *PML-RARA* mutations with targeted deep sequencing using the tailed-PCR method. First-step PCR was performed with target-region sequence specific primers: forward, 5'-TGTTCCAAGCCGCTGT-3' and reverse, 5'-CATCTTCAGCGTGATCA-3'. The 250-base pair of PCR amplicons were used as templates in the second-PCR with the forward primer, 5'-TCGTCGGCAGCGTCAGATGTGTATAAGAGACAGGGGAGCTCATTGAGAAGGTG-3', and reverse primer, 5'-GTCTCGTGGGCTCGGAGATGTGTATAAGAGACAGTTGAGGAGGGTGATTGGTC-3' for indices and the Illumina adaptors using the Nextera Index Kit (Illumina, San Diego, CA). The PCR library was subsequently subjected to sequencing analysis on an Illumina MiSeq sequencer (Illumina). Both ends of the fragments were read by paired-end sequencing, and more than 10,000 reads were analyzed for each fragment. Mutation analysis was performed using the CLC Cancer Research Workbench (QIAGEN). Genetic alterations in the genes frequently altered in myeloid neoplasms were analyzed with the TruSeq custom amplicon sequencing panel and Miseq sequencer according to the manufacturer’s instructions.

### Establishment of wild-type- and mutant *PML-RARA*-expressing HL-60 cells

Human full-length wild-type- (Wt-) and mutant-*PML-RARA* cDNAs were amplified using patient cells, and a FLAG-tag sequence was introduced by PCR. These cDNAs were cloned into the pMX-IP vector (kindly provided by Professor Toshio Kitamura, University of Tokyo, Japan) and transduced into HL-60 cells as described previously [17]. The protein expression of Wt-PML-RARA and mutaunt-PML-RARA was confirmed by Western blot analysis using an anti-FLAG antibody from Sigma-Aldrich [18].

### Antibodies

Anti-FLAG antibody (clone M2) was purchased from Sigma-Aldrich. Anti-mouse horseradish peroxidase antibody was from GE Healthcare (Buckinghamshire, UK). Anti-human CD11b and anti-PML antibodies were from BD Transduction Laboratories (San Jose, CA).

### Cell growth Inhibition assay

Cells were seeded at 1 × 10^4^ / well and cultured in 96-well culture plates for three days. Cell viability was determined using the CellTiter96 Proliferation Assay (Promega, Madison, WI).

### Nitroblue tetrazolium (NBT) reducing ability test

Cells were seeded at 5 × 10^5^ / well and cultured in 12-well culture plates with or without RAs for seven days, and then washed with PBS. The 1:1 mixture of 2 mg/ml of NBT (Sigma Aldrich) and 400 ng/ml of PMA (Wako) was added to the cells, which were then incubated for 20 minutes at 37 °C. After incubation, cells were chilled on ice for five minutes and washed twice with cold PBS. Subsequently, cytospin slides were prepared and 100 cells were counted.

### Immunofluorescence staining

Samples on cytospin slides were fixed with acetone/methanol for 10 minutes at -20°C. After permeabilization with 0.2% Triton X-100 in PBS, the cells were blocked with blocking buffer. Then, they were incubated with primary antibodies, and washed with 0.1% Tween20 (Sigma-Aldrich) in TBS (TBS-T). Subsequently, cells were incubated with secondary antibodies. The cover glasses were mounted on the slides with the DAPI/Antifade reagent, Prolong Gold antifade reagent with DAPI (Invitrogen). Images were acquired using a fluorescence microscopes equipped with digital cameras (Axioskop 2, Zeiss, Oberkochen, Germany) and processed in Axio Vison Rel.4.5 (Zeiss).

### Statistical analysis

All statistical analyses were performed with Graphpad Prism 7 software (GraphPad Software, San Diego, CA), and differences with *P*-values less than 0.05 were considered significant.

## Results

### Clonal expansion of *PML-RARA* mutant cells during APL progression

**Fig 1:**
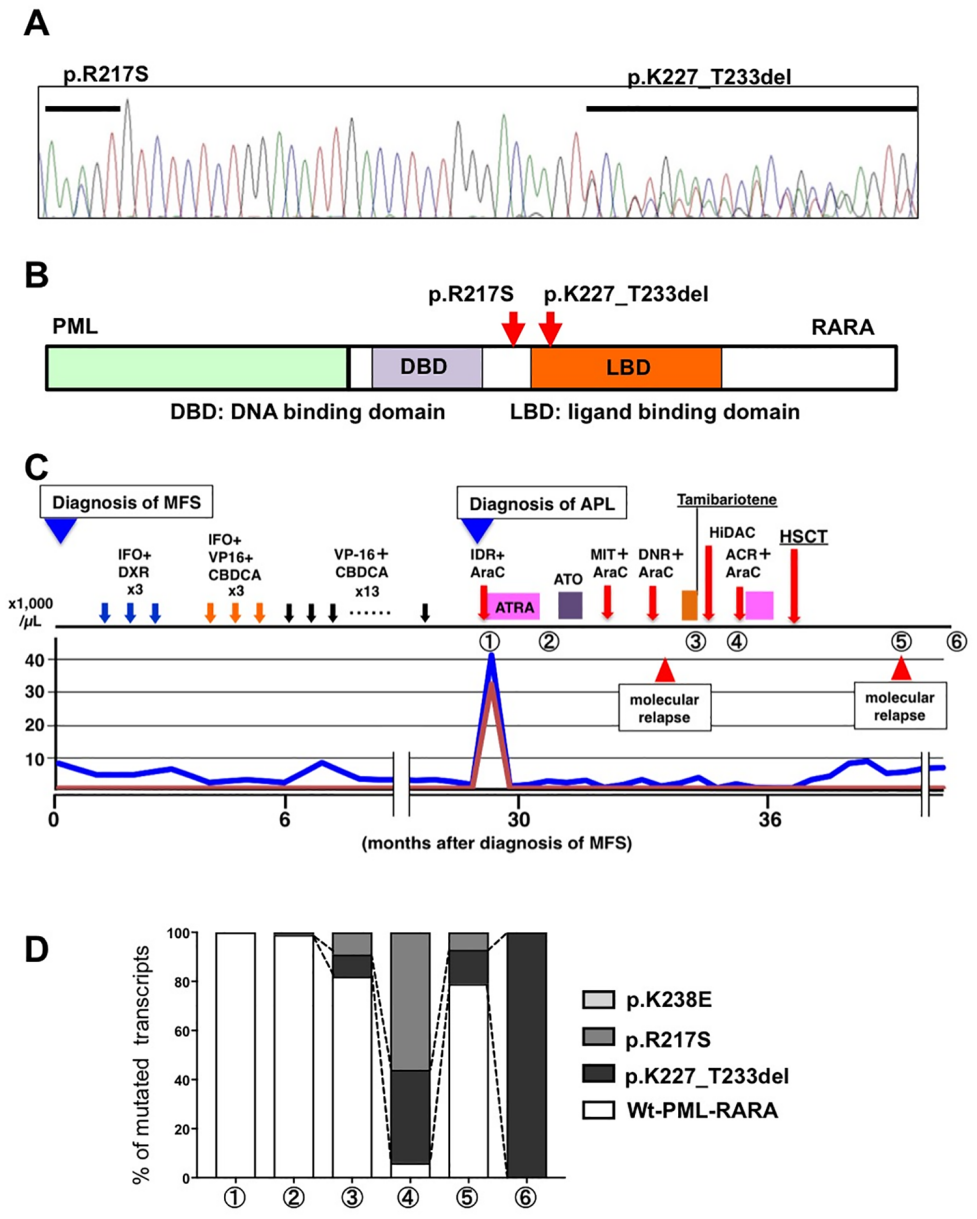
Clonal expansion of *PML-RARA* mutant cells during APL progression. (A) Sequence analyses of the *RARA* region of *PML-RARA* in the RA-resistant patient’s cells. (B) Schematic diagram of PML-RARA. The mutated amino acid residues in the patient’s samples are indicated by red arrows. (C, D) Patient’s clinical course and frequency of mutated *PML-RARA* transcripts in the patient’s samples during APL treatment: at diagnosis ①, after induction therapy ②, at molecular relapse ③, after high-dose cytarabine therapy ④, after allogenic hematopoietic stem cell transplantation ⑤, and at the terminal stage ⑥.

Mutation analysis was performed with BM cells at molecular relapse and we identified two mutations in the *PML-RARA* fusion transcript; a seven amino acid deletion (p.K227_T233del) and a point-mutation p.R217S in the *RARA* region of *PML-RARA* (Fig 1A). These mutated amino acid residues were located in the LBD of the RARA region in the fusion protein (Fig 1B). We confirmed that these mutations occurred in different *PML-RARA* transcripts by the cloning procedure. We retrospectively analyzed the frequency of the identified *PML-RARA* mutations by targeted deep sequencing and confirmed their accumulation during APL progression (Fig 1C and 1D and S1 Table). The total amount of *PML-RARA* transcript increased after re-induction therapy with tamibarotene, and at this point the frequencies of p.K227_T233del and p.R217S were 8.7% and 9.1% of all *PML-RARA* transcripts, respectively (Fig 1C and 1D, point 3). At hematological relapse, these frequencies increased to 37.7% and 56.2%, respectively (Fig 1C and 1D, point 4). Thus, these mutated clones may be associated with RA resistance and therefore selected through the treatment with chemotherapeutic agents and RA. At molecular relapse after HSCT, both p.R217S and p.K227_T233del mutations were identified, but their frequencies had decreased to 7.7% and 13.6%, respectively, and the clone with Wt-*PML-RARA* transcript had again became the dominant clone (Fig 1C and 1D, point 5). Thus, at most of the points we examined, the frequencies of these two mutations were paralleled except at the terminal stage when the APL clone harboring the deletion *PML-RARA* mutation had acquired the p.A216V mutation in the PML region of *PML-RARA* during ATO therapy. This PML mutation is the most common one associated with ATO resistance, and this clone with the deletion mutation comprised 100% of APL cells at the end point (Fig 1C and 1D, point 6) [13, 19].

We also performed targeted sequencing for the 58 genes that were frequently identified in myeloid neoplasms, including the germ-line disposition genes, and found only the *FLT3*-ITD mutation in diagnostic and relapsed samples (S2 Table).

### The deletion mutation abrogates RA-induced APL cell differentiation

**Fig 2:**
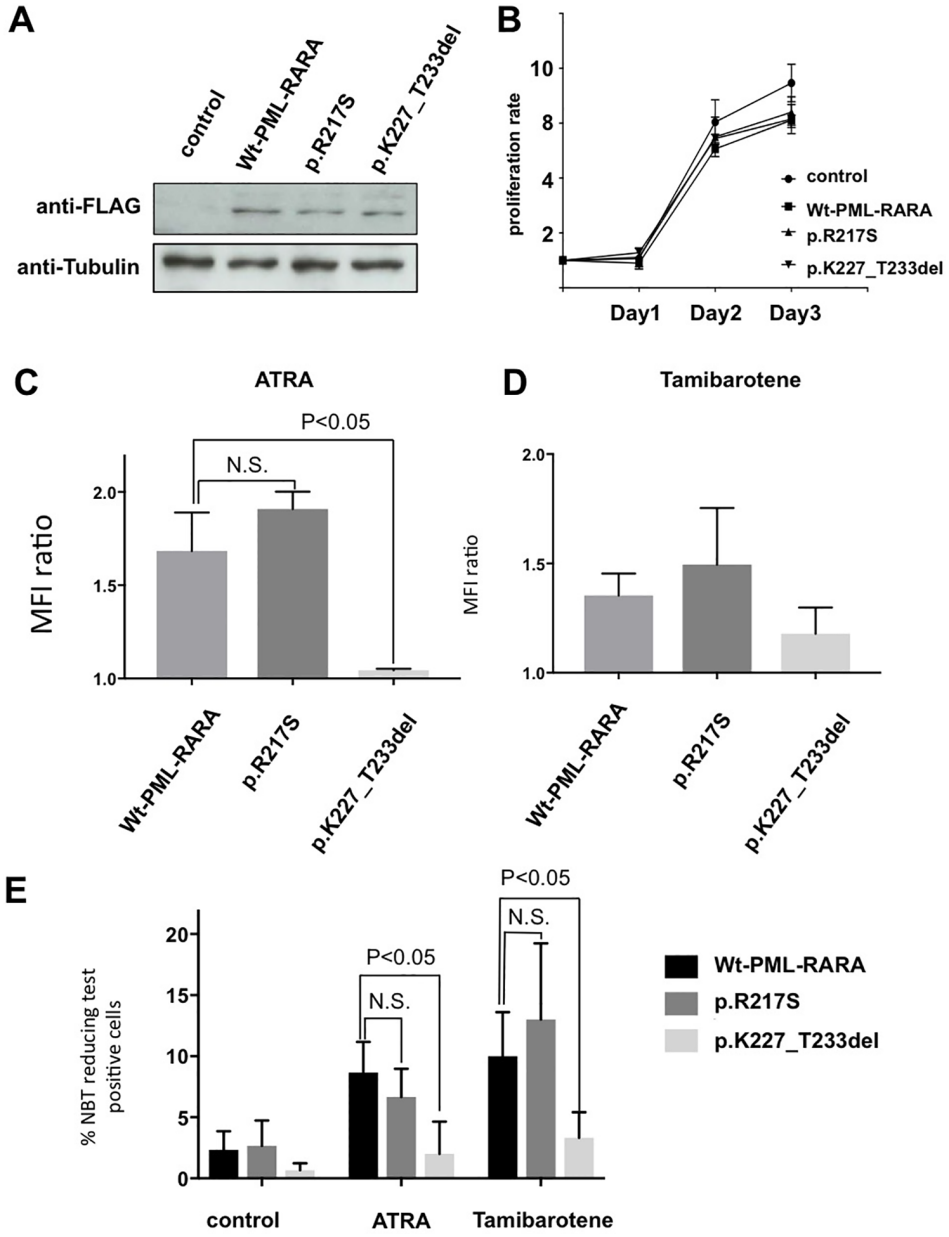
The deletion mutation abrogates RA-induced APL cell differentiation. (A) The expression of PML-RARA protein was examined by Western blot analysis with anti-FLAG antibody in established Wt-*PML-RARA* and mutant- *PML-RARA* expressing cells. (B) Cell growth assay in established Wt-*PML-RARA* and mutant-*PML-RARA* expressing cells. (C, D) Mean fluorescence intensity (MFI) ratios of CD11b to isotype controls in ATRA- (C) and tamibarotene- (D) treated Wt-*PML-RARA* and mutatnt-*PML-RARA* expressing cells. The cells were treated with 1 μM of ATRA or tamibarotene for seven days. Error bars represent mean values ± S.D. of at least three independent experiments. (E) Frequencies of NBT reduction of neutrophils. Cells were treated with 1 μM ATRA or tamibarotene for seven days. Error bars represent mean values ± S.D. of at least three independent experiments.

To determine the impact of these mutations on RA sensitivity, mutated *PML-RARA* was transduced into HL-60 cells, and the protein expression in established cells was confirmed by Western blotting (Fig 2A). Mutant PML/RARA proteins did not have any impact on the growth of the cells in a cell growth assay (Fig 2B). These mutant *PML-RARA*-expressing cells were then treated with ATRA and tamibarotene, and the expression level of CD11b was measured by flow cytometry to evaluate RA-induced differentiation. The relative ratios of mean fluorescence intensity (MFI) of CD11b in ATRA-treated cells to iso-type controls were 1.68, 1.91, and 1.04 for Wt-*PML-RARA-*, p. R217S-, and del p.K227-T233-transduced cells respectively (Fig 2C). CD11b expression level was significantly lower in the deletion mutant-transduced HL-60 cells than that in Wt-*PML-RARA-* and p.R217S-transduced cells (*P* < 0.05). These cells also exhibited the same trend when they were treated with tamibarotene (Fig 2D). Next, we evaluated the RA-induced differentiation ability in these mutant-transduced cells using the NBT reduction test. The frequencies of neutrophil reduction after ATRA and tamibarotene treatment in the Wt-*PML-RARA*, p.R217S-, and del p.K227-T233-transduced cells were 8.7%, 6.7%, and 2.0% with ATRA, and 10.0%, 9.7%, and 3.3% with tamibarotene, respectively. After ATRA and tamibarotene treatment, the deletion mutant-transduced cells had a significantly lower frequency of neutrophil reduction than Wt-*PML-RARA* transduced cells (Fig 2E). Thus, in the deletion mutant-transduced HL-60 cells, CD11b expression and NBT reducing ability after treatment with RA were significantly decreased compared with Wt-*PML-RARA* or p.R217S-*PML-RARA* transduced cells. Collectively, these results indicate that the seven amino acid deletion mutation in LBD confers RA-resistance to APL cells, whereas the p.R217S point mutation does not affect RA sensitivity.

### PML-nuclear body formation is impaired in deletion mutant PML-RARA-expressing cells

**Fig 3:**
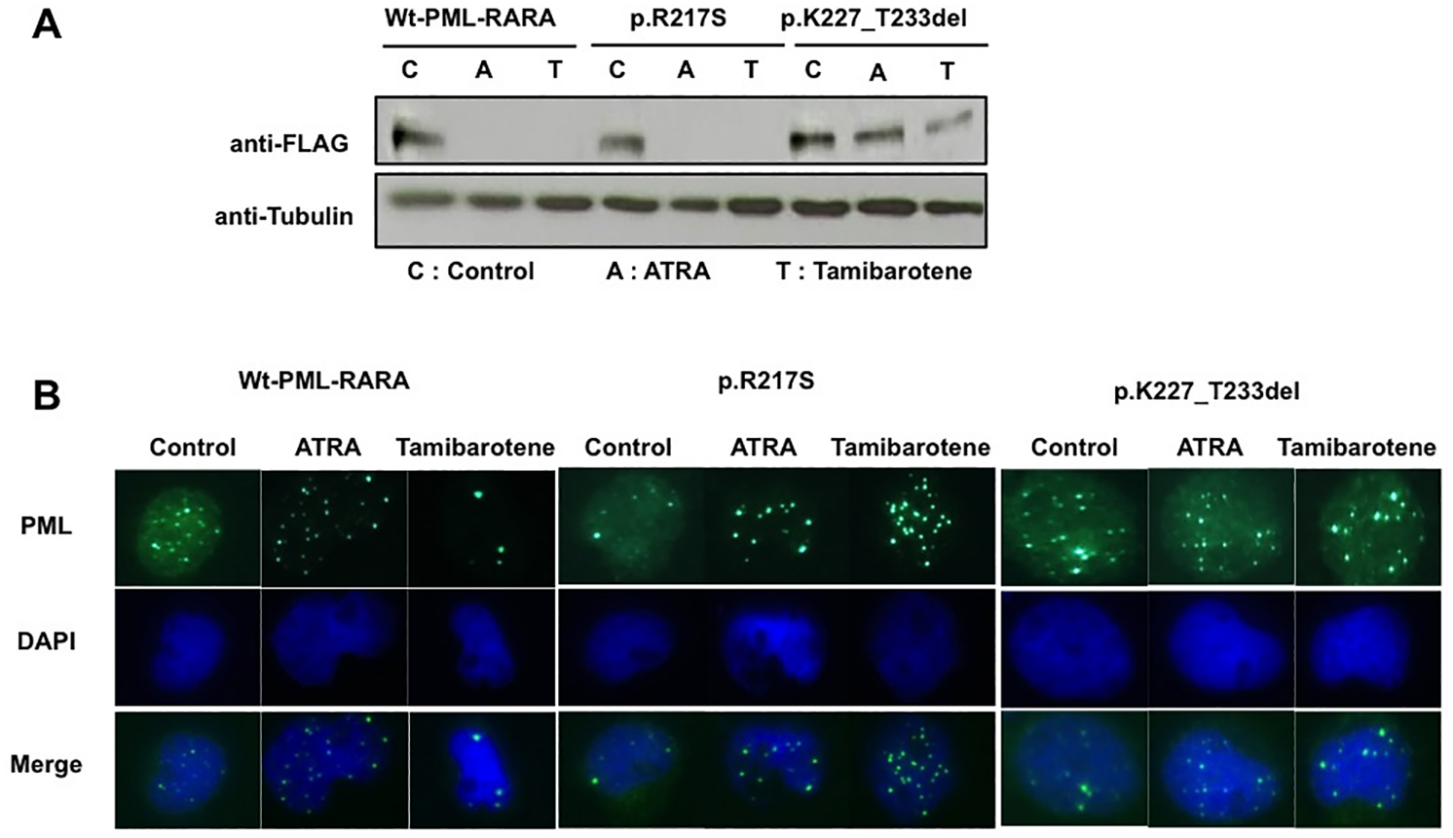
PML-nuclear body formation is impaired in deletion mutant PML-RARA-expressing cells. (A) PML/RARA degradation upon RA treatment in Wt-*PML-RARA-* and mutant-*PML-RARA*-transduced cells was analyzed by Western blot using anti-FLAG antibody. The cells were treated with 1 μM of ATRA or tamibarotene for 12 hours. (B) Localization of PML and PML-nuclear body formation was evaluated by immunofluorescence staining in Wt-*PML-RARA*- and mutated- *PML-RARA*-transduced HL-60 cells. The cells were treated with 1 μM of ATRA or tamibarotene for seven days.

RA is known to degrade PML-RARA and facilitates PML-NB formation in APL cells [20]. Western blotting analysis was performed to determine the extent of PML-RARA degradation upon RA treatment in Wt-*PML-RARA*, and mutant-*PML-RARA* transduced cells, PML/RARA degradation was not observed in p.K227_T233del-transduced cells, indicating that resistance to RA treatment was associated with a lack of PML/RARA degradation (Fig 3A and S1 Fig). We next performed immunofluorescence staining to investigate whether mutant PML-RARA protein abrogates the formation of PML-NBs upon RA treatment and the localization of PMLs was detected using an anti-PML antibody. We confirmed that PML was localized to PML-NBs in a diffuse pattern prior to the RA treatment, and the RA treatment altered the PML staining pattern to a macro-granular pattern in Wt-*PML-RARA*-expressing cells (Fig 3B). Similar results were obtained with p.R217S-*PML-RARA*-expressing cells: PML-NBs with a diffuse pattern in the absence of RA and that became a macrogranular pattern upon RA treatment were observed (Fig 3B). However, p.K227-T233-transduced cells demonstrated a different response to RA treatment and PML-NBs exhibited a diffuse pattern both with and without RA treatment. This was consistent with the observation that the seven amino acid deletion mutation conferred RA-resistance, but that the p.R217S point mutation was not associated with RA resistance *in vitro*. We also examined PML-NB formation in these mutant-*PML-RARA*-expressing cells upon ATO treatment. PML-NBs exhibited a macrogranular pattern in both Wt-*PML-RARA* and mutant-*PML-RARA* transduced cells, and these mutations did not affect ATO-induced PML-NB formation *in vitro* (S2 Fig).

### The deletion mutation alters PML-nuclear body formation with RA in primary APL cells

**Fig 4:**
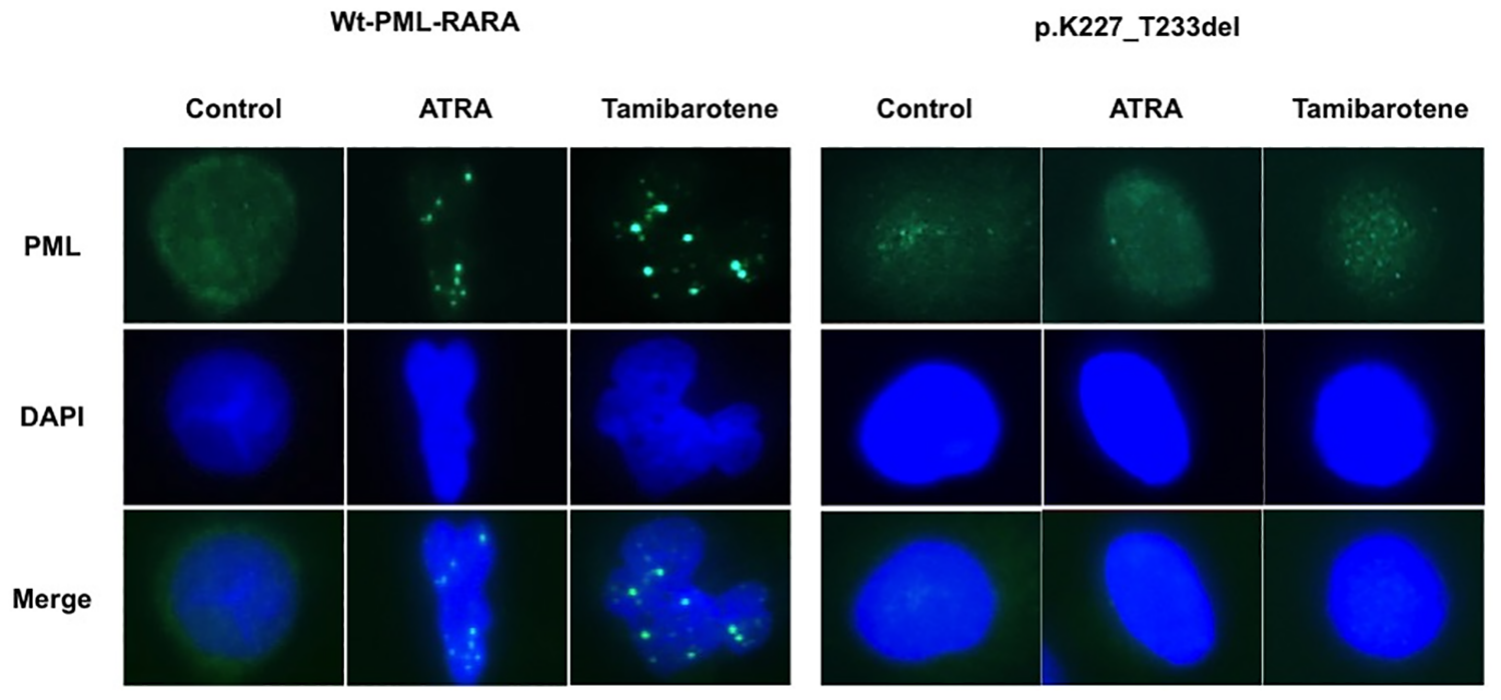
The deletion mutation alters PML-nuclear body formation by RA in primary APL cells. Localization of PML and PML-nuclear body formation was evaluated by Immunofluorescence staining performed with the patient’s APL cells. The cells were treated with 1 μM ATRA or tamibarotene for seven days.

We examined the localization of endogenous Wt-PML-RARA and the deletion mutant-PML-RARA protein and PML-NBs in primary patient APL cells. Primary APL cells at diagnosis and at the terminal stage were subjected to Immunofluorescence staining with anti-PML antibody. As described above, the deletion mutation was found in 100% of the *PML-RARA* transcripts at the end stage. We observed a macrogranular pattern of PML-NBs after both ATRA and tamibarotene treatment at diagnosis, but the staining pattern remained diffuse even after RA treatment at the terminal stage when 100% of the APL cells harbored the deletion mutant (Fig 4). These results suggest that the deletion mutant protein was strongly associated with RA-resistance in the patient’s APL cells.

## Discussion

Our study has demonstrated that a deletion mutation in *PML-RARA* is strongly correlated with resistance to RA therapy. Acquired mutations in *PML-RARA* after RA and/or ATO therapy have been reported in several studies, and are associated with APL progression and resistance to RA and ATO [21–23]. Most mutations previously identified in the *RARA* region in *PML-RARA* were missense mutations, and were located within the LBD. Lou et al. examined *PML-RARA* mutations in 30 RA- and/or ATO-resistant APL patients and identified mutations in 18 patients [14]. In their report, an in-frame deletion mutation was identified in only one patient. Another patient harboring a three amino acid deletion mutation in the LBD domain was reported in 2009 [24]. In our patient, mutations in *PML-RARA* were acquired rapidly three months after induction treatment with ATRA and chemotherapy. These mutated alleles were not identified in the diagnostic BM cells using deep sequencing analysis of *PML-RARA*, indicating that they were either acquired during chemotherapy with ATRA and selected by tamibarotene treatment, or might have already been present at diagnosis at a level lower than our detection sensitivity. The deletion mutation identified in this study resulted in the loss of seven amino acids in the LBD of RARA, that are thought to be crucial for RA binding, and thus abrogated the RA-induced cell differentiation of APL cells. On the other hand, the other mutation at codon 217 in the RARA region of *PML-RARA* was located slightly outside of the LBD and did not alter RA responsiveness *in vitro*. These results indicate that the deletion mutation was more closely associated with the disease progression during RA treatment than the p.R217S mutation. Interestingly, both *PML-RARA* mutant frequencies increased during RA therapy, but only the deletion mutation caused RA resistance *in vitro*.

We also examined the impact of these mutations on the binding ability of ATRA to the RARA region of Wt-PML-RARA and mutated-PML-RARA using three-dimensional model construction of PML-RARA with the Cuemol software (http://www.cuemol.org/en/). The estimated model indicated that the loss of the seven amino acids in the LBD was strongly associated with the loss of binding of ATRA to RARA region, whereas p.R217S had no effect (S3 Fig). Therefore, "*in silico*" models may be useful tools for predicting ATRA sensitivity against mutated PML-RARA.

Therapy-related APL (*t*-APL) occurs recurrently after the chemotherapy with topoisomeraseIIinhibitors such as mitoxantrone and etoposide, and comprises 5–10% of total APLs [25–28]. In contrast to the poor prognosis of therapy-related AML patients, application of ATRA and/or ATO therapy has provided *t*-APL patients with comparable prognoses to primary APL patients [29] [30] [31]. Therefore, predisposition to therapy resistant *t*-APL has not been fully clarified. In this patient, we identified only *FLT3*-ITD mutation among the 58 genes that are frequently mutated in AML. *FLT3*-ITD mutation is also frequent in APL and found in >20% of APL patients. It has been reported that *FLT3*-ITD is associated with high white blood cell counts and high leukemia cells counts in APL, suggesting that it closely relates to APL cell proliferation [32]. Although *FLT3*-ITD is not an independent prognostic factor for APL, this mutation may have been associated with disease progression in this patient.

Taken together, *PML-RARA* mutation is a frequent event in refractory APL patients, and mutation screening is required regardless of therapy duration to determine the ideal therapeutic strategies in these patients. Moreover, these mutations are not the only mechanisms associated with therapy resistance and further investigations are necessary to clarify other resistance mechanisms in APL patients.

## Supporting information

S1 Fig Original western blots used for Fig 3A. (TIF)

S2 Fig PML-nuclear body formation in mutant PML-RARA-expressing cells with ATO treatment. Wt-PML-RARA, p.R217S- and p.K227_T233del-PML-RARA-expressing cells exhibited PML-nuclear body formation with a macrogranular pattern upon ATO treatment. The cells were treated with 10 μM ATO for eight hours.
(TIF)

S3 Fig Estimated binding model of ATRA and PML-RARA *in silico*. The binding ability of ATRA to the RARA region of Wt-PML-RARA and mutated-PML-RARA was estimated with a three-dimensional model construction of PML-RARA using Cuemol software (http://www.cuemol.org/en/).
(TIF)

S1 Table The coverage of *PML-RARA* in targeted deep sequencing. The coverage of the targeted region and read counts of mutated alleles are indicated.
(TIF)

S2 Table Target sequencing panel gene list. List of the 58 genes frequently mutated in myeloid malignancy targeted by deep sequencing.
(TIF)
