# Supplementary figures and images for: Identification of the novel deletion-type PML-RARA mutation associated with the retinoic acid resistance in acute promyelocytic leukemia
Source: PLoS One. 2018 Oct 5;13(10):e0204850. doi: 10.1371/journal.pone.0204850 (PMC6173414; doi:10.1371/journal.pone.0204850)

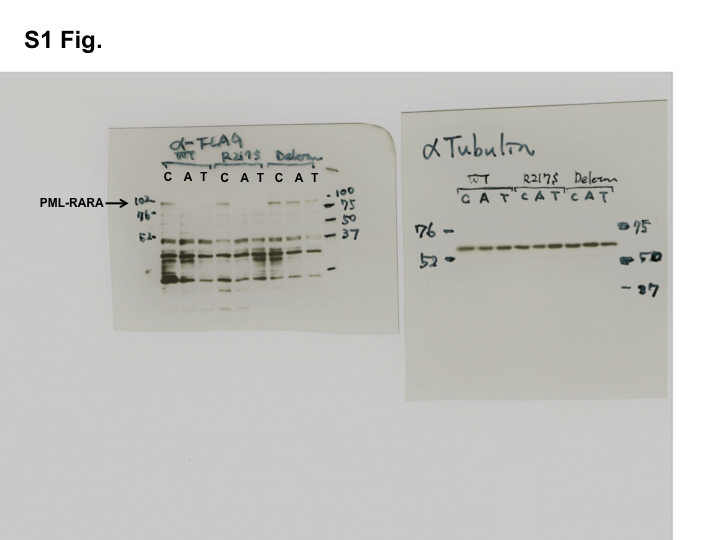

Supplement: S1 Fig — (TIF) [file pone.0204850.s001.tif]

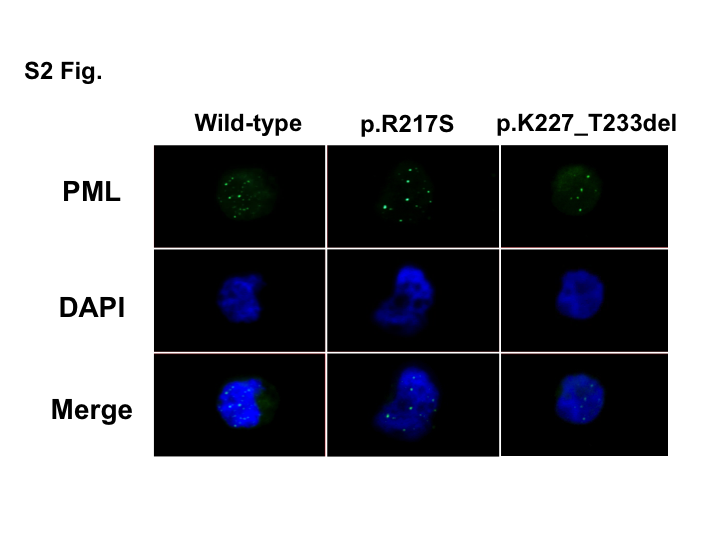

Supplement: S2 Fig — Wt-PML-RARA, p.R217S- and p.K227_T233del-PML-RARA-expressing cells exhibited PML-nuclear body formation with a macrogranular pattern upon ATO treatment. The cells were treated with 10 μM ATO for eight hours. (TIF) [file pone.0204850.s002.tif]

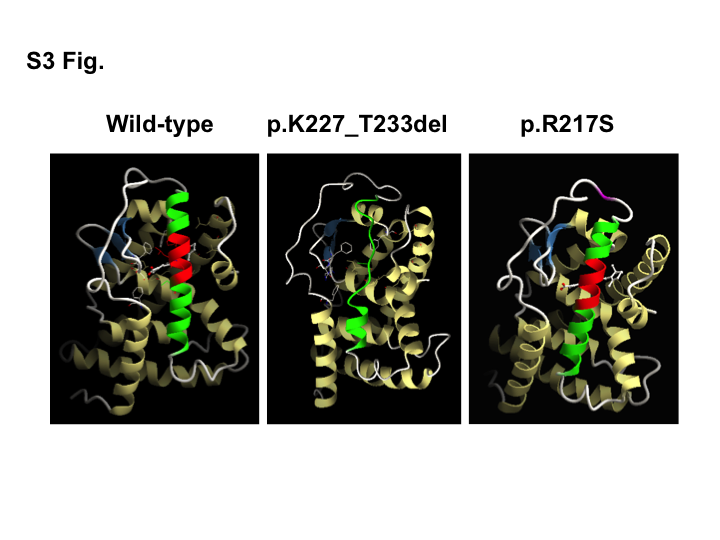

Supplement: S3 Fig — The binding ability of ATRA to the RARA region of Wt-PML-RARA and mutated-PML-RARA was estimated with a three-dimensional model construction of PML-RARA using Cuemol software (http://www.cuemol.org/en/). (TIF) [file pone.0204850.s003.tif]

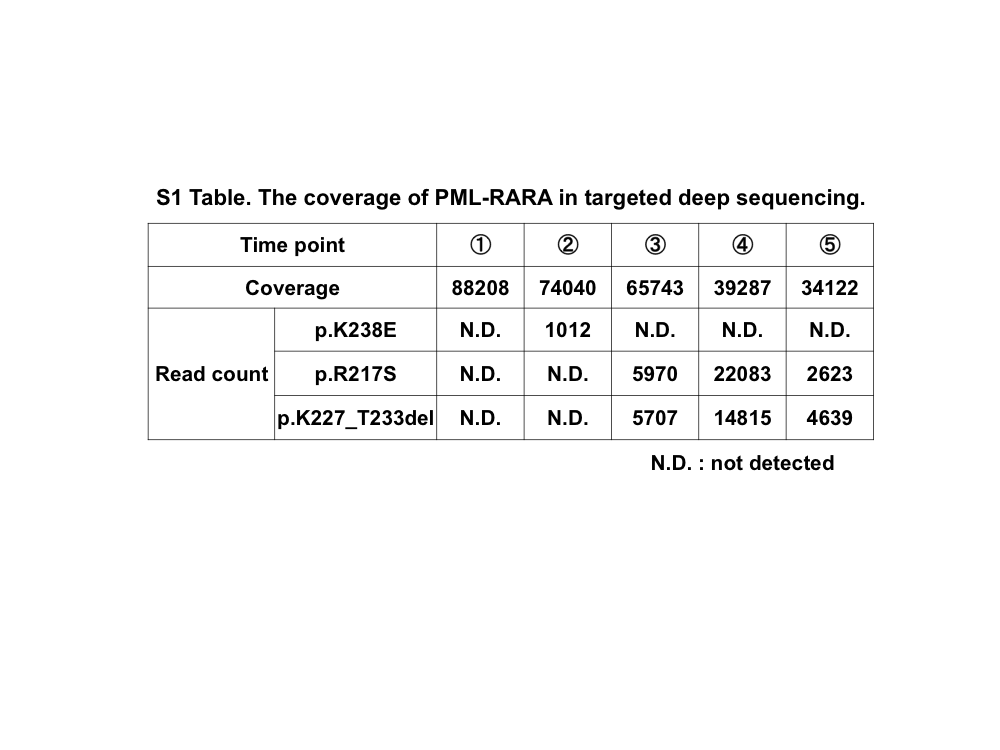

Supplement: S1 Table — The coverage of the targeted region and read counts of mutated alleles are indicated. (TIF) [file pone.0204850.s004.tif]

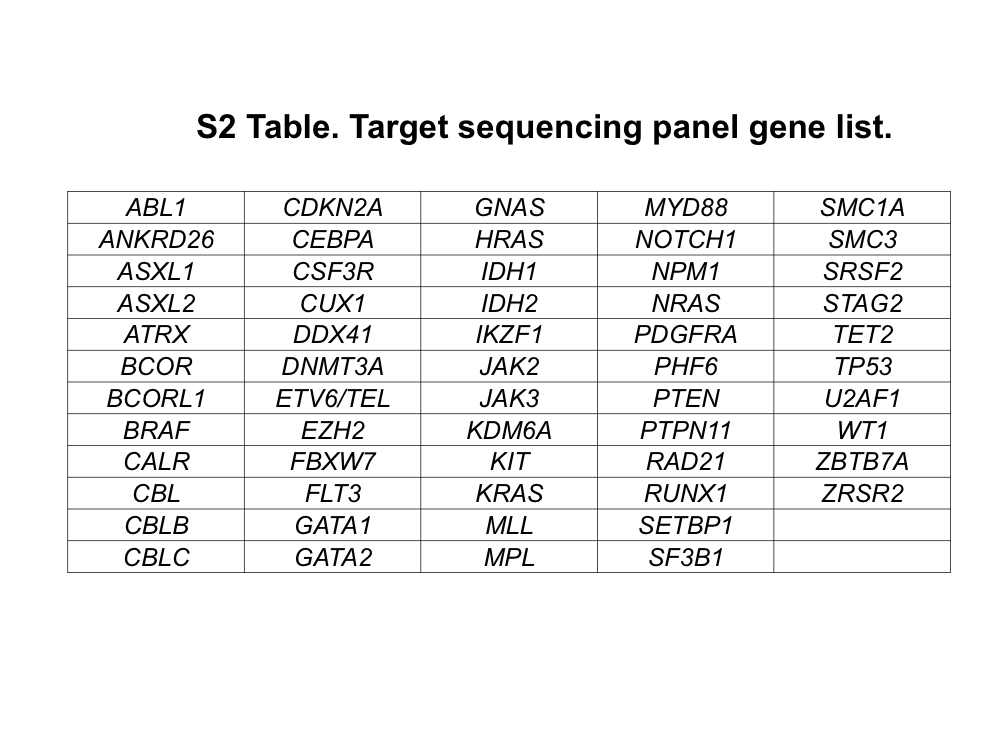

Supplement: S2 Table — List of the 58 genes frequently mutated in myeloid malignancy targeted by deep sequencing. (TIF) [file pone.0204850.s005.tif]
